# Effect of methanol fixation on single-cell RNA sequencing of the murine dentate gyrus

**DOI:** 10.3389/fnmol.2023.1223798

**Published:** 2023-10-04

**Authors:** Marta Sánchez-Carbonell, Patricia Jiménez Peinado, Cathrin Bayer-Kaufmann, Jean-Christopher Hennings, Yvonne Hofmann, Silvio Schmidt, Otto W. Witte, Anja Urbach

**Affiliations:** ^1^Department of Neurology, Jena University Hospital, Jena, Germany; ^2^Institute of Human Genetics, Jena University Hospital, Jena, Germany; ^3^Department of Internal Medicine V, Jena University Hospital, Jena, Germany; ^4^Jena Center for Healthy Aging, Jena University Hospital, Jena, Germany; ^5^Brain Imaging Center, Jena University Hospital, Jena, Germany; ^6^Aging Research Center (ARC) Jena, Jena, Germany

**Keywords:** methanol fixation, primary neural cells, SORT-seq, dentate gyrus, adult neurogenesis, single-cell RNA sequencing

## Abstract

Single-cell RNA sequencing (scRNA-seq) provides a powerful tool to evaluate the transcriptomic landscape and heterogeneity of thousands of cells in parallel. However, complex study designs or the unavailability of in-house instruments require the temporal disconnection between sample preparation and library construction, raising the need for efficient sample preservation methods which are compatible with scRNA-seq downstream analysis. Several studies evaluated the effect of methanol fixation as preservation method, yet none of them deeply assessed its effect on adult primary dissociated brain tissue. Here, we evaluated its effect on murine dentate gyrus (DG) single cell suspensions and on subsequent scRNA-seq downstream analysis by performing SOrting and Robot-assisted Transcriptome SEQuencing (SORT-seq), a partially robotized version of the CEL-seq2 protocol. Our results show that MeOH fixation preserves RNA integrity and has no apparent effects on cDNA library construction. They also suggest that fixation protects from sorting-induced cell stress and increases the proportion of high-quality cells. Despite evidence of mRNA leakage in fixed cells, their relative gene expression levels correlate well with those of fresh cells and fixation does not significantly affect the variance of the dataset. Moreover, it allows the identification of all major DG cell populations, including neural precursors, granule neurons and different glial cell types, with a tendency to preserve more neurons that are underrepresented in fresh samples. Overall, our data show that MeOH fixation is suitable for preserving primary neural cells for subsequent single-cell RNA profiling, helping to overcome challenges arising from complex workflows, improve experimental flexibility and facilitate scientific collaboration.

## 1. Introduction

In recent years, high-throughput single-cell RNA sequencing (scRNA-seq) has emerged as a powerful tool in neuroscience research. By obtaining expression data from thousands of cells in parallel, scRNA-seq provides comprehensive insight into the molecular and cellular diversity of the nervous system, from identifying rare cell populations and subtypes, to studying the dynamic changes in tissue composition and gene expression that occur during development, injury, or disease.

The implementation of such studies often involves complex experimental designs and requires highly specialized instrumentation. These factors can be limiting if the necessary equipment is not available at the time of cell isolation and a temporal disconnection between sample preparation and library construction is required. This calls for appropriate preservation methods that maintain sufficient numbers of high-quality cells without compromising transcriptome analysis. To this end, recent studies have developed various protocols to store and preserve cells for scRNA-seq analysis, including methanol (MeOH) fixation. MeOH is a dehydrating fixative that, at high concentrations, denatures proteins but preserves nucleic acids in a collapsed form, which can be reversed by rehydration (Srinivasan et al., 2002; Hostein et al., 2011). Since its discovery as a single cell preservative for scRNA-seq, it has been used primarily in combination with PBS rehydration (Alles et al., 2017; Wohnhaas et al., 2019; Wang et al., 2021b; Gutiérrez-Franco et al., 2023). In principle, this method has proven successful for the preservation of various cell types for scRNA-seq, for example cells from Drosophila embryos, perinatal mouse brain tissue, hiPSC-derived neural cells, and murine and human cell lines (Alles et al., 2017; Wohnhaas et al., 2019; Wang et al., 2021b; Gutiérrez-Franco et al., 2023). However, depending on the cell or tissue type, it can also introduce artifacts, which may alter the transcriptional profiles or cell composition in the dataset (Wohnhaas et al., 2019; Wang et al., 2021b). In some cases, such as human PBMCs or monocyte-derived macrophages, no cDNA at all could be obtained from PBS-rehydrated MeOH-fixed samples (Chen et al., 2018; Wohnhaas et al., 2019). Subsequent studies noted that the failure in some primary cell types was due to RNA degradation during rehydration in PBS, and developed an improved protocol. They demonstrate that resuspension in 3x saline-sodium citrate buffer (SSC) instead of PBS rehydration significantly improves RNA integrity of sensitive cells and can be successfully used for scRNA-seq of PBMCs, kidney cells, and other primary cell types (Chen et al., 2018; Denisenko et al., 2020). To our knowledge, only one study has examined the effects of MeOH on scRNA-seq of brain tissue (Alles et al., 2017) while another one has applied it to establish the cellular composition of the mouse subventricular zone (Zywitza et al., 2018), both using PBS rehydration. However, while the feasibility study of Alles et al. (2017) showed that MeOH fixation preserves the cellular composition of newborn mouse hindbrain and cerebellum, no conclusions can be drawn about the actual effects of MeOH fixation on subsequent scRNA-seq analysis due to the lack of a freshly prepared control sample.

To address this gap and establish a fixation protocol for large-scale analyses of the adult murine dentate gyrus (DG), we conducted a pilot study in which we adapted the MeOH fixation protocol published by Chen et al. (2018) to preserve primary cells isolated from this area for scRNA-seq. The DG, a part of the hippocampal formation, is a relatively small brain structure with important functions in learning, memory and mood regulation (Scharfman, 2007). It received particular attention with the discovery of its potential to generate new neurons throughout life. This relies on a small population of neural stem cells residing in a specific neurogenic niche that provides the appropriate microenvironment to sustain neurogenesis beyond development (Gonçalves et al., 2016). Compared to other methods for studying adult neurogenesis, such as traditional histology, bulk sequencing or single-cell PCR, scRNA-seq offers several advantages, including unbiased, high-resolution and transcriptome-wide analysis, identification of rare and novel cell types or cell states, and cell–cell interaction analysis. However, transcriptional profiling of the DG neurogenic lineage still faces several challenges. This includes the sparsity of stem cells and their progeny compared to the large number of neurons, calling for enrichment methods or pooling of samples if transitory states or the interaction of stem cells with their niche are to be studied. Inaccessibility of instruments to process samples immediately may be another issue, in particular in complex study designs including multiple time points or interventions. To facilitate such studies, treatments are needed that allow long-term storage of primary neural cells without RNA degradation, maintain the cellular heterogeneity of the niche and preserve the molecular status of the cells as at the time of collection.

To this end, we investigated the suitability of MeOH fixation followed by resuspension in SSC buffer to preserve single-cell suspensions of the mouse DG for subsequent scRNA-seq analysis. Quality assessment was based on several parameters, including RNA integrity, cDNA library complexity, number of transcripts and genes per cell, percentage of mitochondrial genes per cell and stress gene expression, clustering, and cell annotation. We identified great similarities but also some differences between the datasets of fixed and freshly processed cells, which need to be considered but did not affect downstream analysis. Taken together, our results show that MeOH fixation combined with SSC resuspension is an effective method to preserve and recover primary cells isolated from the DG for scRNA-seq if samples cannot be processed immediately.

## 2. Methods

### 2.1. Animals

The studies were carried out on a total of 25 C57BL/6 J mice of mixed gender and age. Mice were kept under specific pathogen free conditions on a 14 h light/10 h dark cycle with food and water *ad libitum*. All animal procedures were in strict compliance with the European animal welfare regulations (EU directive 2010/63/EU and 2007/526/EC guidelines) and the German legislation on the protection of animals, and approved by the local animal welfare committee (TWZ22-2017).

### 2.2. DG dissection and single cell dissociation

**Figure 1:**
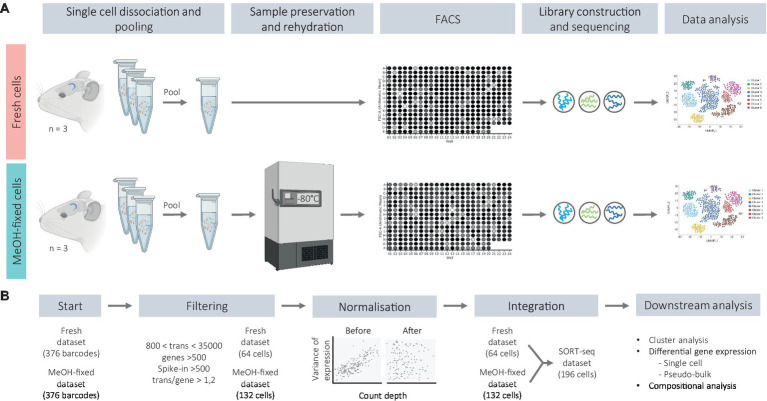
Schematic representation of the experimental workflow **(A)** and bioinformatic analysis **(B)**.

The DGs were dissected as whole mounts from brain hemispheres in PBS with 10 mM glucose. Each DG was individually subjected to an enzymatic and mechanical dissociation according to manufacturer’s protocols (Neural Tissue Dissociation Kit P, Miltenyi Biotec) and single cells were obtained in DPBS without calcium and magnesium containing 0.1 M D-(+)-Trehalose dihydrate at 4°C. From now on, cells were kept on ice. The density and viability of the cell suspensions were evaluated using trypan blue and a hemocytometer. Thereafter, the cell suspensions were pooled at equal ratios as indicated in the next sections, and either MeOH-fixed or immediately processed for RNA quality assessment or for FAC-sorting (Figure 1), which were conducted independently.

### 2.3. MeOH fixation and rehydration of DG cells

MeOH fixation was adapted from a protocol provided by 10x Genomics (CG000136). After dissociation, cells were transferred to 1.5 mL LoBind DNA Eppendorf tubes and pelleted at 300 rcf at 4°C for 5 min. The supernatant was removed carefully and the cell pellet resuspended in one volume of ice-cold DPBS without calcium and magnesium but containing 0.5 M D-(+)-Trehalose dihydrate. Next, four volumes of 100% MeOH pre-chilled to −20°C were added dropwise while gently mixing the cell suspension (final concentration: 10^6^ cells/mL in 20:80 PBS/MeOH 0,1 M Trehalose). The MeOH-fixed cells were kept at −20°C for 30 min and then stored at −80°C for 3 weeks. For rehydration, suspensions were moved from −80 to 4°C on ice and kept for 5 min to equilibrate the temperature. Cells were pelleted at 1,000 rcf at 4°C for 5 min, and resuspended in 3x SSC Buffer with 0.04% BSA, 0.2 U/μL of RNase inhibitor and 1 mM DL-Dithiothreitol. Subsequently, MeOH-fixed cells were processed independently either for RNA quality assessment or for FAC-sorting (see below).

### 2.4. RNA quality assessment

To obtain sufficient numbers of cells for paired measurements on the same sample, i.e., before and after fixation, we pooled cell suspensions (2x from 3 postnatal day 7 animals, and 3x from 4 adult animals, *n* = 5 independent experiments). One third of each pooled cell suspension was immediately used to extract RNA from freshly isolated cells. The two thirds left were MeOH-fixed, stored and rehydrated 3 weeks later for RNA quality assessment. The RNA was extracted using 1:5 Chloroform-QIAzol, precipitated overnight at −20°C after adding 0.16-fold volumes sodium acetate (2 M pH 4.0) and 1.1-fold volumes isopropanol to the aqueous phase, washed with 75% ethanol and dissolved in DNase/RNase-Free Distilled Water. RNA quality was assessed with Agilent RNA 6000 Nano Kit on an Agilent 2,100 Bioanalyzer (Agilent Technologies, Santa Clara, CA).

### 2.5. Fluorescence-activated cell sorting

Fresh and MeOH-fixed cells were processed using the plate-based SOrting and Robot-assisted Transcriptome sequencing (SORT-seq; Muraro et al., 2016) method at Single Cell Discoveries (Utrecht, Netherlands).

After rehydration of the pooled MeOH-fixed cells (n = 1 from 4 mice pooled), the single cell suspension was centrifuged at 300 rcf at 4°C for 5 min and cells were resuspended in ice-cold DPBS without calcium and magnesium containing 5% HEPES. Next, single cells were FAC-sorted into 384-well microplates containing well-specific barcoded primers, ERCC spike-ins and reverse transcription reagents using a FACSAria™ Fusion cell sorter (BD Biosciences). Wells O21-O24 and P21-P24 were left empty as a no-template control. Different forward and side-scatter parameters were used for gating to discriminate single cells from debris and doublets or aggregates (Supplementary Figure S1). To have the lowest bias between samples, control cell suspensions from age-matched mice were freshly prepared, pooled and FAC-sorted the same day into another 384-well cell capture microplate (*n* = 1 from 3 mice pooled). In this case, Sytox AADvanced™ Dead Cell Stain was added (5 min, at 4°C, in the dark) prior to FAC-sorting to ensure that only viable cells are sorted. The filled microplates were sealed and centrifuged at 2,000 rcf for 2 min at 4°C, snap-frozen on dry ice and stored at −80°C.

### 2.6. Library preparation and sequencing

Microplates containing FAC-sorted cells from fresh and MeOH-fixed samples were shipped on dry ice to Single Cell Discoveries and libraries were prepared according to the CEL-Seq2 protocol (Hashimshony et al., 2016; Muraro et al., 2016). After cell lysis at 65°C, the RNA was reverse transcribed into cDNA followed by second-strand cDNA synthesis. The barcoded cDNA of all wells from one plate was pooled and amplified by *In Vitro* Transcription. The amplified RNA was fragmented, reverse transcribed into cDNA and again amplified using TruSeq small RNA primers (Illumina) to generate the final sequencing libraries. The quality and yield of the amplified RNA and the final cDNA libraries were checked on an Agilent Bioanalyzer. Thereafter, the libraries were paired-end sequenced on an Illumina Nextseq™ 500, high output, with a 1 × 75 bp Illumina kit (R1: 26 cycles, index read: 6 cycles, R2: 60 cycles) aiming at 75000 reads/cell. Raw sequencing data were mapped to the GRCm38 mouse reference genome and preliminary count matrices were generated for each sample using STAR (Spliced Transcripts Alignment to a Reference; dimension of the matrices: number of barcodes x number of transcripts). Briefly, read 1 was used for assigning reads to correct cells and libraries, read 2 was mapped to gene models and reads that mapped equally well to multiple genomic locations were discarded. Read counts were then corrected for amplification bias by using the UMI barcodes. Reads that had identical combinations of library, cellular, and molecular barcodes and were mapped to the same gene were considered duplicates and were removed. The number of UMIs per transcript was counted for each cell barcode, followed by adjusting the transcript counts to the expected number of molecules based on counts, 4,096 possible UMIs and poissonian counting statistics.

### 2.7. Pre-processing of scRNA-seq data

Bioinformatic analysis was conducted using R programming language (R v4.2.0). Raw count matrices were pre-processed using the Seurat package v4.1.0. Firstly, ERCC RNA spike-in number of counts were removed from the count matrices and added into the metadata from the Seurat object. Barcodes with total transcript counts between 800 and 35,000, more than 500 total genes, a transcript to gene ratio greater than 1.2 and more than 500 ERCC counts were considered to contain successfully captured healthy single cells and were positively selected in the filtering step. This resulted in 64 fresh cells and 132 MeOH-fixed cells in the filtered dataset. After removing low quality barcodes, samples were normalized by regularized negative binomial regression (SCTransform) and integrated using the top 2,000 variable genes (Stuart et al., 2019). Based on the elbow plot after principal component analysis (PCA), the top 12 principal components (PCs) were selected for dimensionality reduction and graph-based clustering, and the first 15 PCs were applied for Uniform Manifold Approximation and Projection (UMAP) visualization. The marker genes of each cluster were identified with a Wilcoxon rank sum test implemented in the “FindAllMarkers” Seurat function with the following criteria: adjusted value of *p* <0.05 (FDR correction), log-fold change ≥0.5 and ratio of the detection fraction between the cluster and the rest (pct.1/pct.2) > 2.5.

### 2.8. Stress gene selection

In order to evaluate the stress level of the dissociated and sorted cells, we analyzed the expression of genes coding for transcription factors, signal transducers, heat shock proteins and pro-apoptotic proteins that become rapidly upregulated in the brain under stress conditions resembling those to be expected during cell isolation and sorting, i.e., cell dissociation (van den Brink et al., 2017; Denisenko et al., 2020), traumatic brain injury and ischemia (Urbach et al., 2006; Sieber et al., 2014; Meng et al., 2017). This list comprised *Atf3*, *ARC*, *Casp1*, *Casp9*, *Egr1*, *Hsp90aa1*, *Hsp90ab1*, *Hspa1a*, *Hspa1b*, *Hspa8*, *Hspb1*, *Hspe1*, *Hsph1*, *Ier3*, *Junb*, *REM2*, *Socs3*.

### 2.9. Assessment of ambient RNA contamination

To estimate the levels of non-endogenous, ambient RNA as indicator of mRNA leakage, we applied a tool originally invented to decontaminate droplet-based scRNA-seq data from background RNA (DecontX; Yang et al., 2020). The raw count matrices of each condition after filtering served as input and the raw unfiltered count matrix as background parameter.

### 2.10. Evaluation of dropouts based on expression level

To determine whether MeOH fixation leads to a loss of transcripts depending on their expression level, we calculated the number of dropout events across the entire transcriptome (Wang et al., 2021b). Therefore, we established a set of increasing gene expression level thresholds, applied them on the raw count matrix at a single cell level, and quantified the number of genes with expression levels equal and above each individual threshold for each condition.

### 2.11. Evaluation of gene expression depending on transcript length

The mus_musculus.GRCm38.102 GTF-file was used to calculate the non-overlapping exon length per gene using the package GenomicFeatures (v1.50.4). Gene names were annotated into gene symbol annotation using the package AnnotationDbi (v1.60.2). Pseudo-bulk datasets were generated by averaging the expression profiles of all cells per condition using the AverageExpression function from Seurat and merged with the exon length information. The genes were sorted into 20 different groups based on their length, ensuring that each group contained the same number of genes. Afterwards, the ratio between gene expression levels was calculated per gene by dividing the expression levels in fresh cells by those in fixed cells. Genes were sorted in the same groups as before and the ratios were compared between length groups.

### 2.12. Correlation and hierarchical cluster analysis

Pairwise correlation matrices of all filtered cells were calculated, from either the raw counts or the Pearson’s residuals obtained after SCTransform normalization, with package stats (v4.3.0). To plot these correlation results and evaluate the impact of MeOH on clustering, the package pheatmap (v1.0.12) was applied.

### 2.13. Statistical analysis

Statistical analyses were performed with the package stats (v4.3.0). The Shapiro Wilk test was used to assess normality. Wilcoxon rank-Sum tests for unpaired or paired samples, in cease of RNA integrity experiments, were applied to assess differences between MeOH-fixed and fresh cells, and *p*-values were corrected by false discovery rate (FDR) when multiple comparisons were performed. *p*-values and adjusted *p*-values < 0.05 were considered statistically significant.

## 3. Results

### 3.1. MeOH fixation preserves RNA integrity and has no detectable effect on cDNA library construction

**Figure 2:**
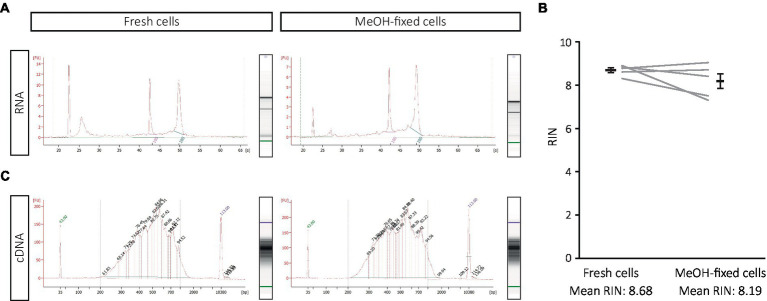
Effect of MeOH fixation on RNA quality and cDNA library construction from DG samples. **(A,B)** RNA integrity from freshly dissociated and MeOH-fixed cells after SSC-based rehydration, with **(A)** showing a representative electropherogram of one of the five the experiments displayed in **(B)**. **(C)** Electropherograms of the cDNA libraries from freshly dissociated and MeOH-fixed cells. Statistics: Shapiro Wilk test for normality and Wilcoxon test for paired samples.

MeOH is a fixative that dehydrates cells, preserves the nucleic acids in a collapsed form and can be completely removed by SSC-based rehydration (Chen et al., 2018). To evaluate the effect of this procedure on transcriptome analysis of DG cells, we first compared the quality of the RNA isolated from MeOH-fixed-rehydrated and fresh cells as well as the fragment size distribution in cDNA libraries before sequencing. RNA of equally high quality could be extracted from both, fresh and MeOH-fixed cells (RIN mean > 8; Figures 2A,B). Likewise, the electropherograms of the cDNA libraries from MeOH-fixed and fresh cells appeared highly similar (fresh vs. MeOH: cDNA conc.: 3,115 vs. 2,992 pg./μL; av. size: 511 vs. 510 bp; Figure 2C). These results suggest that high quality RNA, and cDNA libraries of similar complexity to fresh cells can be obtained from MeOH-fixed cells.

### 3.2. MeOH fixation reduces stress arising from microfluidic single cell capture

**Figure 3:**
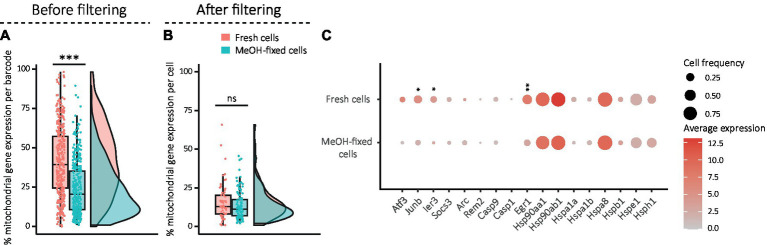
Evaluation of damage and stress signatures in fresh and MeOH-fixed DG cells. **(A,B)** Combination of box and density plots showing the percentage of mitochondrial gene expression **(A)** per barcode before filtering (fresh: mean: 41%, median: 39%; MeOH: mean: 20%, median: 21%) and **(B)** per cell after filtering (fresh: mean: 15.9%, median: 13.9%; MeOH: mean: 13.6%, median: 11%), horizontal lines indicating the median. **(C)** Dotplot showing the average expression of stress gene markers after filtering. The circles’ areas from the dotplot are proportional to the frequency of cells with expression >0 within the condition and the color encodes the average expression level across all cells within the condition. Statistics: **(A–C)** Shapiro Wilk test for normality and Wilcoxon rank-Sum test for unpaired samples (***p < 0.001) with FDR correction in **(C)**; *adj. *p* < 0.05, **adj. *p* < 0.01, ***adj. *p* < 0.001.

Single-cell RNA sequencing (scRNA-seq) uses fluorescence-activated cell sorting (FACS) or microfluidic-based instruments such as the Fluidigm C1 or the 10x Chromium controller to capture single cells into individual reaction containers. Even if the processes have been highly optimized, the pressure and shear stress during FACS isolation could negatively affect the quality of sorted cells, potentially impacting downstream analysis. Similarly, long capture times and exposure to hostile buffers during droplet encapsulation may alter the physiological states of the cells. Here, we evaluated the expression level of mitochondrially encoded genes as a sign of stressed, poor-quality and damaged cells, where a high percentage of mitochondrial gene expression per barcode was considered indicative (Chen et al., 2018; Osorio and Cai, 2021). The percentage of mitochondrial gene expression was calculated and compared between conditions before filtering of the dataset to analyze the state of all sorted cells (barcodes), and after filtering to see if there were still differences in the barcodes considered as good quality cells. Filtering consisted in retaining all barcodes that had between 800 and 35,000 transcripts, more than 500 genes, more than 500 spike-in transcripts and a ratio of transcript per gene greater than 1.2. Before filtering, barcodes of MeOH-fixed samples comprised a significantly lower proportion of mitochondrial gene expression than those of fresh cells (Figure 3A). Filtering significantly reduced the percentage of mitochondrial genes in the dataset, although it was not used as filtering criterion, suggesting that it successfully removed low quality cells (Figure 3B). Moreover, the differences between fresh and fixed cells decreased to a level that was not statistically significant (Figure 3B). We next analyzed the expression levels of individual stress-related immediate early genes to assess whether traces of stress remained in the filtered dataset. This revealed that three stress genes (Figure 3C) were significantly less expressed in MeOH-fixed cells than in fresh cells, indicating that MeOH fixation mitigates single cell capture-induced stress response.

### 3.3. MeOH-fixed samples possess a higher fraction of good quality cells but show signs of RNA leakage

**Figure 4:**
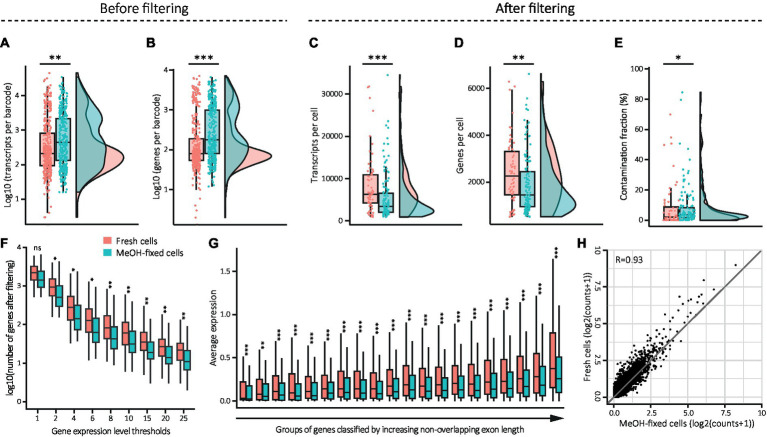
Effect of MeOH fixation on gene expression values from DG cells. **(A–E)** Combination of box and density plots showing the distribution of transcripts (**A**, fresh: mean: 1,782, median: 209; MeOH: mean: 2,134, median: 444) and genes per barcode (**B**, fresh: mean: 497, median: 80; MeOH: mean: 740, median: 178) before quality control filtering, and the distribution of transcripts (**C**, fresh: mean: 8,476, median: 6,332; MeOH: mean: 5,569, median: 3,463), genes (**D**, fresh: mean: 2,395, median: 2,259; MeOH: mean: 1,895, median: 1,449) and ambient RNA contamination fraction (**E**, fresh: mean: 0.082, median: 0.020; MeOH: mean: 0.083, median: 0.035) in cells remaining after filtering, horizontal lines indicating the median. **(F)** Effect of MeOH fixation on gene detection depending on their expression level. Nine gene expression thresholds were applied on the raw matrix of counts. Each category on the x axis represents the sum of all genes with an expression level equal and above the defined threshold. **(G)** Comparison of average detection levels of genes depending on their length, from left to right (in bp): 66–1,272, 1,272–1701, 1702–2083, 2084–2,419, 2,419–2,724, 2,724–3,031, 3,033–3,341, 3,342–3,652, 3,652–3,995, 3,995–4,358, 4,358–4,733, 4,734–5,193, 5,194–5,631, 5,633–6,129, 6,130–6,772, 6,774–7,556, 7,556–8,514, 8,514–9,857, 9,859–12,513 and 12,520–117,334. **(H)** Pseudo-bulk gene expression correlation of MeOH-fixed and fresh cells. Statistics: **(A–G)** Shapiro Wilk test for normality and Wilcoxon rank-Sum test for unpaired samples (*p < 0.05, **p < 0.01, ***p < 0.001) with FDR correction in **(F,G)** (*adj. *p* < 0.05, **adj. *p* < 0.01, ***adj. *p* < 0.001). **(H)** Pearson’s correlation test.

Organic fixatives such as MeOH can compromise the cell membrane structure, leading to the loss of cytoplasmic mRNAs (Hobro and Smith, 2017). To assess the quality of the samples immediately after sorting, and to detect any possible indications of general leakage, we compared the transcripts and genes per barcode before quality control filtering. Our analysis revealed that the MeOH-fixed sample had significantly higher numbers of transcripts and genes per barcode than the fresh sample (Figures 4A,B), indicating that, in general, the MeOH-fixed sample comprised a larger number of high-quality cells in comparison to the fresh sample. As expected, a greater number of cells survived the filtering step in the MeOH-fixed sample (*n* = 132 out of 378 cells) than in the fresh one (*n* = 64 out of 378 cells). Surprisingly, the remaining high-quality fixed cells exhibited significantly lower numbers of transcripts and genes in contrast to fresh cells (Figures 4C,D). This indicates a possible mRNA leakage in MeOH-fixed cells, which might have been obscured by the substantial proportion of low-quality fresh cells in the unfiltered dataset. To investigate the possibility of RNA leakage further, we applied a tool developed to decontaminate scRNA-seq datasets from ambient RNA (DecontX). Ambient RNA typically originates from leaky cell membranes or cells damaged during dissociation, and can therefore be considered as indication of RNA leakage. The detected amounts of contaminating mRNA suggest that MeOH-fixed cells contained more ambient mRNA than fresh cells (Figure 4E; ~1.5% median difference), supporting our assumption of mRNA leakage in fixed cells.

### 3.4. Relative gene expression levels of fixed cells correlate well with those of fresh cells

We next tested whether this apparent leakage affects transcripts depending on their expression level or depending on their length, and thus may introduce bias into downstream analysis. To do so, we first compared the number of dropout events across the entire transcriptome. This was achieved by setting a series of increasing thresholds for the gene expression level in the raw count matrix at the single cell level and assessing the number of genes remaining as the threshold increased (Figure 4F). Regardless of the expression threshold, we detected fewer genes in MeOH-fixed cells compared to fresh cells, indicating that MeOH fixation leads to a loss of transcripts independently of their abundance. Next, we determined if the leakage affected long and short transcripts differently by classifying groups of genes based on their exon length and comparing their group average expression levels. This revealed that MeOH-fixed cells had significantly fewer transcripts in each gene length group (Figure 4G). To assess if the fixed cells had a lower number of transcripts regardless of gene length, we calculated the expression ratio for each gene by dividing the value in fresh cells by that in fixed cells and sorted the genes into the same gene length groups as before. This showed that the ratio increments significantly as the gene length increases, indicating that the differences in expression levels between conditions become greater with increasing gene length (Supplementary Figure S2 and Supplementary Table S1). Finally, we checked if MeOH fixation changes overall gene expression levels. Therefore, we performed a correlation analysis on pseudo-bulk gene expression profiles derived from fresh and fixed samples after filtering. This revealed a strong positive linear correlation between the two sample types (Pearson’s correlation coefficient of *r* = 0.93), indicating that the transcriptome of MeOH-fixed cells is highly similar to that of fresh cells (Figure 4H).

Collectively, these findings indicate that although MeOH fixation may cause RNA leakage and a slightly higher tendency toward the loss of longer transcripts, it does not introduce significant bias in gene expression levels.

### 3.5. Clustering and cell annotation remain unaffected after MeOH fixation, while cell type composition changes

**Figure 5:**
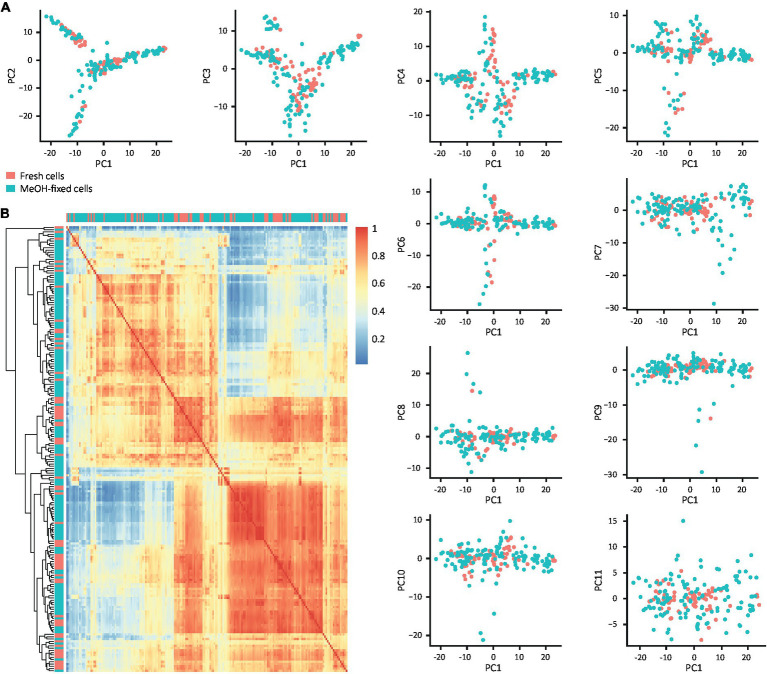
Effect of MeOH fixation on the transcriptome profiles of filtered DG cells. **(A)** Scatter plots of the first 11 PCs confronted with PC1. **(B)** Heat map of pairwise correlation coefficients of the raw count matrix for fixed and fresh cells. Cells in the heat map are ordered according to clustering based on the Euclidean distance of the correlation coefficients. The color scale indicates the degree of correlation (blue, low correlation; red, high correlation) and the labels represent the individual cells.

To explore if MeOH fixation affects scRNA-seq downstream analysis and biological inference, we evaluated its effect on the transcriptome profile and cell type composition from DG samples. Firstly, we assessed the impact of fixation on the variance in their transcriptomic profiles by applying principal component analysis (PCA) and testing whether fixation explains the variance in the first 11 principal components (PCs). The scatter plots of PC1 against PC2 to PC11 showed that the cells were distributed independently of their treatment (Figure 5A), indicating that fixation is not a source of variation and confirming that fixed and fresh cells have similar expression patterns. To verify these results, we calculated the pairwise correlation matrix from the raw counts and grouped the cells according to the Euclidean distance between their correlation factors, allowing comparison of fixed and fresh cells in all dimensions of their transcriptome regardless of cell identity (Figure 5B). Consistent with the previous result, no high correlation spot formed by only fresh or fixed cells could be identified and no cells clustered by condition were observed, indicating that MeOH fixation is not affecting the transcriptome profile of the cells.

**Figure 6:**
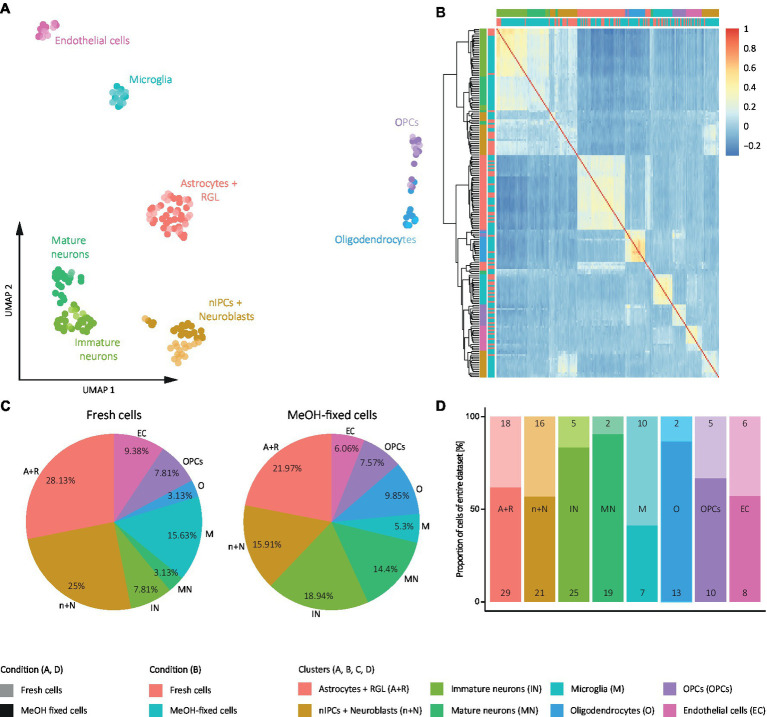
Analysis of effect of MeOH on DG cell identities from SORT-seq experiment. **(A)** UMAP representation of the clustered cells. **(B)** Heat map of the pairwise correlation coefficients calculated with Pearson residuals from regularized negative binomial regression of gene expression for fixed and fresh cells. Cells in the heat map are ordered according to clustering based on the Euclidean distance of the correlation coefficients. The color scale indicates the degree of correlation (blue, low correlation; red, high correlation). **(C)** Pie graphs representing the % of each cell identity in fresh cells and MeOH-fixed samples. **(D)** Bar plot representing the composition of each cluster by MeOH-fixed cells and fresh cells. Numbers inside the bar plots represent the absolute number of fixed and fresh cells in each cluster. **(A,C,D)** Color code: colored by cell identity and shaded by condition. **(B)** Color code: colored by cell identity and by condition. Pink: Fresh cells; Blue: MeOH-fixed cells.

Secondly, to study the effect of MeOH fixation on cell type composition, we performed graph-based clustering of the cells and identified the cell identities based on the results of differential gene expression analysis between each cluster and all remaining cells. This revealed 8 distinct clusters representing the major DG cell populations showing similarity between conditions (Figure 6A), i.e., astrocytes and radial glia-like cells (RGLs; *S100β*, *Sox2*, *Sox9*, *Aldoc*, *Hopx*, and *Id4*), neural intermediate progenitor cells (nIPCs) and neuroblasts (*Eomes*, *DCX*, *Ccnd2*, *Sox11*, *Calb2*), immature neurons (*Dsp., Gda*, *Rbm24*, *Rasgrf2*, *Rbfox3*, and *Ncam1*), mature neurons (*Rasgrp1*, *Smad3*, *Kcnip3*, *Mfsd4*, and *Tanc1*), microglia (*Csf1r* and *Cx3cr1*), oligodendrocytes (*Mbp* and *Plp1*) oligodendrocyte progenitor cells (OPCs; *Oligo1*, *Sox10*, and *Cspg4*) and endothelial cells (*Prom1* and *Esam*).

To investigate whether any of these cell populations was particularly affected by MeOH fixation, we calculated the proportions of each cell identity in datasets of MeOH-fixed and fresh cells and the frequency of fixed and fresh cells within each cell identity. This showed that both conditions differed in terms of their cell identity composition (Figure 6C) and that most of the cell identities comprised more fixed than fresh cells (Figure 6D). This was particularly evident for neurons, indicating that MeOH may protect them from sorting-induced stress. As a consequence, a decrease in the proportions in the other cell identities was also observed (Figure 6C). Supporting those results, when evaluating both conditions together (Figure 6D), we observed that the identities belonging to neuronal clusters consisted of a higher percentage of fixed than fresh cells.

Based on these findings, we examined whether MeOH fixation compromises the transcriptome profile of any of the cell identities in particular. The same approach used to test its influence on the whole sample was followed, but calculating the pairwise correlation matrix and clustering from Pearson residuals rather than from raw counts (Figure 6B). This revealed that cells within each cell population were not ordered by condition, nor was there a spot of high correlation in the heat map that exclusively included fixed cells or fresh cells, which rather appeared intermixed. An exception were the nIPCs, for which the dendrogram demonstrated a separation into two clusters of which one contained almost only fixed cells. However, the correlation coefficients of cells within each cluster and those of cells between these clusters were equivalent. A slight separation of fresh and fixed nIPCs was also observable in the UMAP representation (Figure 6A), though their transcriptomes are similar enough to be combined in one cluster as revealed by graph-based clustering (Figure 6A). To examine the possibility of a fixation effect in nIPCs arising from the original matrix of counts, we isolated this population, calculated the correlation matrix of their raw transcriptomes and represented it in a heat map (Supplementary Figure S5). Here, neither the clustering nor the correlation factors between cells depended on whether the cells were fixed or not. However, as could be expected the correlation coefficients of the cells varied to some extent, presumably due to the fact that this population comprises various cell stages and states (early and committed cells in the process of division and/or differentiation). These results, together with the UMAP representation (Figure 6A), indicate that MeOH fixation does not define cell clustering within each cell identity, although we cannot completely rule out minor effects on nIPCs. Therefore, despite influencing cell identity proportions, putatively due to the preservation of otherwise fragile neurons, MeOH fixation does not particularly affect the transcriptome profile of any of the DG cell identities identified.

### 3.6. MeOH fixation shows similar effects in droplet-based scRNA-seq

To verify these data and to investigate whether MeOH fixation works similarly well on microfluidic droplet-based platforms, we compared an in-house dataset of fixed DG cells to an age and tissue-matched dataset from fresh cells published in Hochgerner et al. (2018) (GSE95315; dataset A). Since the datasets were generated in different laboratories, using different 10x Genomics chemistry versions and sequencing technologies, and varied in total cell number, the analysis was performed using approaches that are less influenced by the number of transcripts and the absolute number of cells. First, as for the SORT-seq experiment, we inferred how fixation affects the transcriptomic profiles by PCA, and evaluated the first 11 PCs that capture the largest amount of variation in the dataset. The scatter plots of PC1 versus PCs 2–11 show that cells do not cluster depending on condition, indicating that fixation does not explain the variance within the first 11 PCs (Supplementary Figure S3). Subsequently, for further insight on the findings about cell taxonomy found in the analysis of the sorted cells, data were grouped using graph-based clustering. We could identify 13 distinct clusters, all of which were composed of fresh and MeOH-fixed cells (Supplementary Figures 4A,B). In addition to the cell types detected in the smaller SORT-seq experiment (section 3.5), we detected clusters representing Mossy cells (*Rab6b* and *Cck*), GABA neurons (*Gad2* and *Nxph1*), Cajal Retzius cells (*Reln*, *Ndnf*, and *Lhx5*), as well as separate clusters of astrocytes (*S100β*, *Sox2*, *Aqp4*, and *Id3*) and RGLs (*Lpar1*, *Sox9*, and *Hopx*), which clustered together in the small SORT-seq experiment. To assess whether MeOH fixation affects the cellular composition of the sample, i.e., the ratios of cell populations, we calculated the proportions of each cell identity in both datasets. Similar to the SORT-seq experiment, we observed a shift in cell proportions upon MeOH fixation that was primarily related to an increase in dentate granule neuron numbers (Supplementary Figure S4C).

Collectively, the results from the integrated 10x Genomics datasets support the conclusions derived from the SORT-seq experiment. Specifically, they confirm that MeOH fixation is not a major source of variation in the data, that it allows the identification of the same cell types as in datasets of fresh DG samples, and that it protects dentate granule neurons.

## 4. Discussion

Single-cell RNA-seq (scRNA-seq) has emerged as a powerful tool for studying cellular heterogeneity and gene expression in neuroscience. However, despite its potential, it still faces limitations, especially when it comes to preserving dissociated cells for downstream analyses. Sample preservation is critical to any study design that requires a temporal decoupling between sample preparation and library construction. Key challenges in this context include maintaining the integrity of the cells and RNA, avoiding transcriptional loss and interference with library assembly, and preserving the transcriptional profile of each cell and the original cell type composition. Several protocols have been developed for this purpose, such as cryopreservation, formaldehyde crosslinking, and MeOH fixation, with success rates varying both between and within protocols. While MeOH fixation was successfully used in scRNA profiling of several cell lines, it introduced biases of varying degrees in transcriptome profiling of others, as well as of primary cells and tissues (Alles et al., 2017; Chen et al., 2018; Zywitza et al., 2018; Wohnhaas et al., 2019; Denisenko et al., 2020; Wang et al., 2021b). The discrepancies between study outcomes can be attributed to differences in sample types, processing methods, and recovery protocols. For example, PBS-rehydrated MeOH-fixed immune cells suffered from RNA degradation due to reactivation of endogenous RNAses, which can be avoided by resuspending fixed cells in high salt buffers such as 3x SSC (Chen et al., 2018). Other complications include RNA leakage leading to an increase in ambient RNA and dropout of low expression genes, and the inability to preserve certain cell types (Alles et al., 2017; Chen et al., 2018; Zywitza et al., 2018; Wohnhaas et al., 2019; Denisenko et al., 2020; Wang et al., 2021b). Consequently, MeOH, while compatible in principle with scRNA-seq, may introduce cell- and tissue-specific complications that must be carefully determined for each new cell type or target tissue.

We here show that MeOH fixation combined with SSC-based rehydration is compatible with sorting- or droplet-based scRNA-seq of adult primary neural tissue. Compared to fresh samples, MeOH fixation has no effect on RNA integrity and library size distribution. Moreover, our data suggest that it prevents sorting-induced cell stress, increases the yield of high-quality cells and preserves cell types that are particularly vulnerable to *ex vivo* stress. Importantly, despite evidence of mRNA leakage from fixed cells, fixation does not significantly bias the datasets. Rather, the relative gene expression levels from MeOH-fixed cells correlate well with those from fresh samples, and MeOH fixation had no apparent effects on the variance of the dataset or subsequent clustering, cell annotation and cell-type composition.

To study the single cell transcriptomes of highly complex tissues, cells need to be dissociated and captured for barcoding and sequencing. This may damage isolated cells, trigger stress responses and alter their gene expression, especially in more vulnerable cell types such as those derived from adult brain. Several results from our study confirm such biases and show that they are mitigated by MeOH fixation. Our finding that barcodes from fresh cells before filtering contained fewer transcripts and genes and a higher percentage of mitochondrially encoded genes suggests that more fresh than fixed cells were damaged by sorting and consequently lost cytoplasmic RNA (Ilicic et al., 2016). Although excessive RNA degradation could explain part of these findings, it is unlikely to be a major factor in our samples, as both fresh and fixed cells had comparably high RNA quality. The higher stress signatures maintained in the quality-filtered fresh cells further confirm their susceptibility to sample processing and demonstrate that fixation prevents sorting-induced stress of primary neural cells (Lopez and Hulspas, 2020). This was to be expected since the freshly isolated cells were still alive at the time of capture, as ensured by our Sytox-based sorting strategy, while fixation leads to immediate arrest of any transcriptional activity. These factors ultimately translated into a higher yield of high-quality cells in the fixed sample after filtering, which affected almost every cell type in our dataset. The most affected were neurons, a cell type that is particularly susceptible to stressors such as hypoxia and oxidative stress (Cobley et al., 2018), which indicates that the presented protocol is beneficial for the preservation of fragile cell types for scRNA-seq from brain. Currently, there is only one other study that analyzed the number of high-quality cells in MeOH-fixed/SSC-recovered primary samples (Denisenko et al., 2020). In contrast to us, they found no differences between datasets of fixed and freshly prepared kidney cells in this respect. This could be due to the different nature and resilience of the tissues analyzed, as kidney cells may be more resistant to pressure and stress of sorting (Kalogeris et al., 2012). Yet, their study confirms that fixation does not negatively affect the quality of primary cell suspensions.

Organic fixatives such as MeOH can increase membrane permeability and may lead to leakage of cytoplasmic mRNAs into the extracellular milieu, which could increase noise in the data due to ambient RNA contamination (Hobro and Smith, 2017). Indeed, we found several indications for mRNA leakage from MeOH-fixed DG cells, such as a lower number of transcripts and genes per cell as well as higher proportions of ambient RNA and dropout events after filtering. This was consistent with findings in human PBMCs and mouse kidney cells preserved in the same way as our cells (Chen et al., 2018; Denisenko et al., 2020). Importantly, leakage did not result in a skewed representation of low- and high-expressed genes as observed in PBS-rehydrated MeOH-fixed cell lines (Wang et al., 2021b). In addition, leakage may not be the only cause of reduced transcript counts in fixed cells. Evidence suggests that longer transcripts are less likely to recover after fixation due to their higher structural complexity and greater stability, which may compromise their amplification (Trotta, 2014). Our evaluation of MeOH effects as a function of non-overlapping exon length supports this assumption and shows a stronger underrepresentation of longer transcripts compared to short ones in fixed samples, similar to MeOH-fixed cell lines rehydrated in PBS (Wang et al., 2021b). This could be a drawback and must be considered in scRNA-seq studies targeting long transcripts or splice variants, for which fresh samples would be more advisable. Despite these potential confounding factors, we found no evidence of adverse effects of MeOH fixation on the transcriptional profile of our samples. Clustering, pseudo-bulk expression as well as principal component analysis confirmed a high correlation between both datasets. Importantly, we identified the same cell types in both fixed and fresh samples, both, in our relatively small dataset with only 372 input cells per condition as well as in the 10x dataset with several thousands of cells. There was no evidence for a depletion of particular cell types as has been observed in datasets of MeOH-fixed PBS-rehydrated primary cells (Alles et al., 2017; Chen et al., 2018; Wohnhaas et al., 2019; Gutiérrez-Franco et al., 2023). Instead, most cell populations contained even more fixed than fresh cells. This was particularly noticeable for dentate granule neurons, the most abundant cell type in the DG, whose number was lower in fresh samples of both, the SORT-seq and 10x experiment. Moreover, we observed that neurons are clearly underrepresented in the SORT-seq compared to the 10x dataset. This might be explained by differences in cell dissociation protocols between both experiments or by the greater mechanical stress on cells during FAC-sorting compared to the encapsulation in the 10x Chromium Controller, which might particularly affect delicate cell types such as granule neurons (Wang et al., 2021a). At the moment, there is one other study examining cell proportions in MeOH-fixed primary brain samples, i.e., such extracted from the subventricular zone (Zywitza et al., 2018). In contrast to us, they found no differences in cell type composition of fixed and freshly dissociated cells. This could be due of the different cell composition of the two brain regions: while neurons are the most common cell type of the DG, none are found in the subventricular zone. Altogether, the findings from both our experiments suggest that MeOH fixation in combination with SSC resuspension maintains cell type composition in scRNA-seq datasets of brain samples in a more physiological manner.

In summary, our study determined the suitability of MeOH fixation combined with SSC buffer reconstitution for the preservation of adult neural cells. We show that fixation does not induce systematic bias in terms of the cells transcriptomic profiles or cell type composition in the resulting dataset, and even preserves cells at higher quality and numbers compared to fresh cells. We also identify possible confounding factors that might be relevant under certain scenarios and need to be considered when planning experiments, including the leakage of RNA and a tendentially greater loss of longer transcripts. Based on our results, we consider MeOH fixation with SSC-based rehydration as a highly feasible preservation method for primary neural samples when direct processing of fresh samples is not possible. This allows experiments to be performed when instruments for library generation are not directly accessible and facilitates experiments with complex workflows, such as those studying developmental and aging processes or those that require sample pooling to obtain sufficient numbers of rare cells.

## 5. Limitations

The SORT-seq data shown in this study is based on a small-scale experiment that includes one technical replicate per condition, which consisted of 3–4 pooled DGs from different mice. To increase the robustness of the findings, we included droplet-based datasets consisting of several thousands of cells. While our results indicate that MeOH fixation combined with reconstitution in SSC buffer has no major effect on the transcriptomic profile of adult mouse DG cells, we found indications for systematic bias in the data, such as RNA leakage, reduced signatures of stress and cellular damage, and a protection of mature granule neurons. Researchers should take all these factors into account while planning and optimizing their experiments. Furthermore, it is important to point out that MeOH fixation can result in weakening or even disappearance of fluorescence signals (Schwarz et al., 2015; Kirschnick et al., 2021). Therefore, if the purpose of a study is the enrichment of specific cell populations based on fluorescent reporter protein expression and FACS, MeOH fixation might not be the method of choice.

## Data availability statement

The datasets presented in this study can be found in online repositories. The names of the repository/repositories and accession number(s) can be found at: https://www.ncbi.nlm.nih.gov/geo/, GSE232052; https://www.ncbi.nlm.nih.gov/geo/, GSE241452.

## Ethics statement

The animal study was approved by Jena local animal welfare committee (TWZ22-2017). The study was conducted in accordance with the local legislation and institutional requirements.

## Author contributions

MS-C and PJP: conceptualization, methodology, formal analysis and writing—original draft, review and editing. CB-K, J-CH, and YH: methodology. SS: conceptualization. OW: resources and supervision. AU: conceptualization, methodology, validation, writing—review and editing, funding acquisition, resources and supervision. All authors contributed to the article and approved the submitted version.
