# Supplementary material for: Effect of methanol fixation on single-cell RNA sequencing of the murine dentate gyrus
Source: Front Mol Neurosci. 2023 Oct 4;16:1223798. doi: 10.3389/fnmol.2023.1223798 (PMC10582346; doi:10.3389/fnmol.2023.1223798)
Supplement: Supplementary file 1 [file Data_Sheet_1.pdf]

Supplemental material: Effect of methanol fixation on single cell RNA sequencing of the murine dentate gyrus

# 1. Supplementary methods

## 1.1 Animals

The studies were carried out on a total of 25 C57BL/6J mice of mixed gender and age. Mice were kept under specific pathogen free conditions on a 14 hours light/10 hours dark cycle with food and water ad libitum. All animal procedures were in strict compliance with the European animal welfare regulations (EU directive 2010/63/EU and 2007/526/EC guidelines) and the German legislation on the protection of animals, and approved by the local animal welfare committee (TWZ22-2017).

## 1.2 (Dentate gyrus) DG dissection, single cell dissociation and fixation for droplet-based scRNA-seq of MeOH-fixed cells

The DGs of 5 mice (C57Bl/6J background, mixed gender, age: 4 weeks) were dissected as whole mounts in HBSS with calcium, magnesium and 10 mM glucose. Each DG was individually subjected to enzymatic and mechanical dissociation according to manufacturer's protocols (Papain Dissociation System, Worthington kit). Single cells were obtained in HBSS with calcium, magnesium and 0.1 M D-(+)-Trehalose dihydrate at 4 °C and kept on ice. After evaluation of the density and confirmation of a viability of at least 85 %, the cells were fixed in MeOH as described in section 2.3 and stored at -80 °C.

## 1.3 Library preparation and sequencing of MeOH-fixed cells by droplet-based scRNA-seq

The fixed cell suspensions were shipped on dry ice to Single Cell Discoveries (…). Prior to loading the 10x Chromium chip, cells were rehydrated in SSC buffer as described in section 2.3, filtered, counted and pooled at equal ratios. Cells were loaded at a concentration of 700-1200 cells/µl on a 10x Genomics chip aiming for 10000 cells, and the chip was run on a Chromium controller to initiate GEM formation. Library preparation was performed following standard 10x Genomics guidelines 3' v3.1 workflow with feature barcoding (CG000317 guide) to generate the cDNA library, which was paired-end sequenced on Illumina NovaSeq S4 Flow Cell chip on a NextSeq6000 platform (read 1: 28 cycles, index reads: 2×10 cycles, read 2: 90 cycles) to obtain an average of 70000 reads per cell. FASTQ files were generated from BCL files with Cell Ranger (Zheng et al., 2017), mkfastq 6.0. Read 1 was used for identification of cell barcodes and UMIs. Read 2 was used to map to the GRCm38 mouse reference genome. Filtering of empty barcodes was done following standard Cell Ranger procedures.

## 1.4 Pre-processing of 10x Genomics scRNA-seq data

Similar to the bioinformatic analysis of the SORT-seq data, pre-processing of droplet-based datasets was conducted using R (v4.2.0) and the Seurat package v4.1.0. Briefly, for the MeOH-fixed sample, the raw count matrix was decontaminated using DecontX (Yang et al., 2020) and barcodes were considered high-quality cells when their total transcript counts were between 1000 and 35000, total genes more than 1000, mitochondrial genes less than 15% and a transcript to gene ratio greater than 1.2. Doublets were removed by applying scDblFinder algorithm (Germain et al., 2021) obtaining 5122 high-quality singlets.

An age-matched dataset of fresh cells was extracted from "Dataset A" published in Hochgerner et al., 2018 (GSE95315). This comprised 1063 quality control filtered DG cells from their P24 group. To analyze both datasets together, they were integrated using the top 2000 variable genes (Stuart et al., 2019). Based on the elbow plot after principal component analysis (PCA), the top 15 PCs were selected to describe the merged dataset, and utilized for UMAP representation and graph-based clustering. The Seurat function 'FindAllMarkers' was applied (adjusted p-value < 0.05 (FDR correction), log-fold change ≥ 0.5 and ratio of the detection fraction between the cluster and the rest (pct.1/pct.2) > 2.5) in order to find markers differentially expressed in each cluster to match them to cell types.

## 2. Suppl Figure legends

**Supplementary figure 1. FACS gating strategy for the isolation of murine DG cell populations and distribution of the finally sorted cells.**

(**A**, **B**) Scatter-plots showing the gating strategy to exclude debris based on size (SSC-A) and granularity (FSC-A) for fresh (**A**) and MeOH-fixed (**B**) cells, respectively. (**C-F**) Gating strategy to exclude doublets in fresh (**C**) and MeOH-fixed (**D**) cells based on SSC-H vs. SSC-A scatter-plots followed by a second round of doublet exclusion based on FSC-H vs. FSC-W scatter-plots for both preparations (fresh cells (**E**), MeOH-fixed cells (**F**)). (**G**) Exclusion of dead singlets in fresh cell preparation based on Sytox AADvanced™ Dead Cell Stain. Black dots represent the cells finally collected in the 384-well plates. The plot's titles refer to the parent gate.

**Supplementary figure 2. Ratio of average gene detection levels between fresh and fixed cells as a function of gene length.**

From left to right [in bp]: 66-1272, 1272-1701, 1702-2083, 2084-2419, 2419-2724, 2724-3031, 3033-3341, 3342-3652, 3652-3995, 3995-4358, 4358-4733, 4734-5193, 5194-5631, 5633-6129, 6130-6772, 6774-7556, 7556-8514, 8514-9857, 9859-12513 and 12520-117334. Horizontal line positioned at $y = 1$. Statistics can be found in Supplementary table 1.

**Supplementary figure 3. Effect of MeOH fixation on droplet-based scRNA-seq variance.** Scatter plots of the first 11 PCs confronted with PC1.

**Supplementary figure 4. Analysis of the MeOH effect on DG cell identities from droplet-based scRNA-seq.**

(A-B) UMAP representations of the cells colored by preservation method (A) and by cell identity (B). (C) Pie graphs representing the relative cell type composition in the datasets of fresh and MeOH fixed samples.

**Supplementary figure 5. Effect of MeOH fixation on the raw transcriptome of nIPCs.**

Heat map of pairwise correlation coefficients of the raw count matrix for fixed and fresh nIPCs. Cells were ordered based on the Euclidean distance of the correlation coefficients. The color scale indicates the degree of correlation (blue, low correlation; red, high correlation) and the labels represent the individual cells.

## 1 Supplementary tables text

**Supplementary table 1:** Pairwise comparisons for ratio values represented on Supp. Figure 2. Columns and rows represent the groups of genes classified by increasing non-overlapping exon length, from shortest to longest [in bp]: 66-1272, 1272-1701, 1702-2083, 2084-2419, 2419-2724, 2724-3031,

3033-3341, 3342-3652, 3652-3995, 3995-4358, 4358-4733, 4734-5193, 5194-5631, 5633-6129, 6130-6772, 6774-7556, 7556-8514, 8514-9857, 9859-12513 and 12520-117334. Statistics: Shapiro Wilk test for normality and Wilcoxon rank-Sum test for unpaired samples with FDR correction (light green: adj. $p<0.05$, medium green: adj. $p<0.01$, dark green adj. $p<0.001$).

Groups of genes classified by increasing non-overlapping exon length

| Groups of genes classified by increasing non-overlapping exon length | 66-1272 | 1272-1701 | 1702-2083 | 2084-2419 | 2419-2724 | 2724-3031 | 3033-3341 | 3342-3652 | 3652-3995 | 3995-4358 | 4358-4733 | 4734-5193 | 5194-5631 | 5633-6129 | 6130-6772 | 6774-7556 | 7556-8514 | 8514-9857 | 9859-12513 |
|---|---|---|---|---|---|---|---|---|---|---|---|---|---|---|---|---|---|---|---|
| 1272-1701 | 0,259 | | | | | | | | | | | | | | | | | | |
| 1702-2083 | 0,004 | 0,114 | | | | | | | | | | | | | | | | | |
| 2084-2419 | 0,050 | 0,509 | 0,352 | | | | | | | | | | | | | | | | |
| 2419-2724 | 0,006 | 0,191 | 0,767 | 0,487 | | | | | | | | | | | | | | | |
| 2724-3031 | 0,026 | 0,352 | 0,555 | 0,707 | 0,787 | | | | | | | | | | | | | | |
| 3033-3341 | 0,000 | 0,014 | 0,432 | 0,062 | 0,236 | 0,169 | | | | | | | | | | | | | |
| 3342-3652 | 0,005 | 0,125 | 0,988 | 0,333 | 0,707 | 0,555 | 0,452 | | | | | | | | | | | | |
| 3652-3995 | 0,001 | 0,041 | 0,707 | 0,160 | 0,452 | 0,362 | 0,651 | 0,786 | | | | | | | | | | | |
| 3995-4358 | 0,000 | 0,000 | 0,026 | 0,001 | 0,007 | 0,005 | 0,176 | 0,036 | 0,060 | | | | | | | | | | |
| 4358-4733 | 0,000 | 0,001 | 0,121 | 0,008 | 0,042 | 0,031 | 0,452 | 0,137 | 0,212 | 0,555 | | | | | | | | | |
| 4734-5193 | 0,000 | 0,004 | 0,208 | 0,020 | 0,091 | 0,061 | 0,654 | 0,233 | 0,328 | 0,393 | 0,787 | | | | | | | | |
| 5194-5631 | 0,000 | 0,003 | 0,162 | 0,014 | 0,059 | 0,041 | 0,487 | 0,160 | 0,238 | 0,555 | 0,994 | 0,789 | | | | | | | |
| 5633-6129 | 0,000 | 0,000 | 0,014 | 0,000 | 0,003 | 0,002 | 0,095 | 0,016 | 0,026 | 0,707 | 0,352 | 0,229 | 0,363 | | | | | | |
| 6130-6772 | 0,000 | 0,000 | 0,016 | 0,000 | 0,004 | 0,003 | 0,121 | 0,022 | 0,035 | 0,817 | 0,452 | 0,303 | 0,450 | 0,859 | | | | | |
| 6774-7556 | 0,000 | 0,000 | 0,004 | 0,000 | 0,001 | 0,001 | 0,039 | 0,005 | 0,007 | 0,450 | 0,182 | 0,103 | 0,196 | 0,707 | 0,555 | | | | |
| 7556-8514 | 0,000 | 0,000 | 0,000 | 0,000 | 0,000 | 0,000 | 0,000 | 0,000 | 0,000 | 0,013 | 0,002 | 0,001 | 0,003 | 0,041 | 0,020 | 0,112 | | | |
| 8514-9857 | 0,000 | 0,000 | 0,002 | 0,000 | 0,000 | 0,000 | 0,016 | 0,002 | 0,003 | 0,252 | 0,092 | 0,048 | 0,104 | 0,450 | 0,352 | 0,687 | 0,254 | | |
| 9859-12513 | 0,000 | 0,000 | 0,000 | 0,000 | 0,000 | 0,000 | 0,000 | 0,000 | 0,000 | 0,001 | 0,000 | 0,000 | 0,000 | 0,003 | 0,002 | 0,012 | 0,352 | 0,041 | |
| 12520-117334 | 0,000 | 0,000 | 0,000 | 0,000 | 0,000 | 0,000 | 0,000 | 0,000 | 0,000 | 0,000 | 0,000 | 0,000 | 0,000 | 0,000 | 0,000 | 0,000 | 0,000 | 0,000 | 0,000 |
