# Supplementary figures and images for: Effect of methanol fixation on single-cell RNA sequencing of the murine dentate gyrus
Source: Front Mol Neurosci. 2023 Oct 4;16:1223798. doi: 10.3389/fnmol.2023.1223798 (PMC10582346; doi:10.3389/fnmol.2023.1223798)

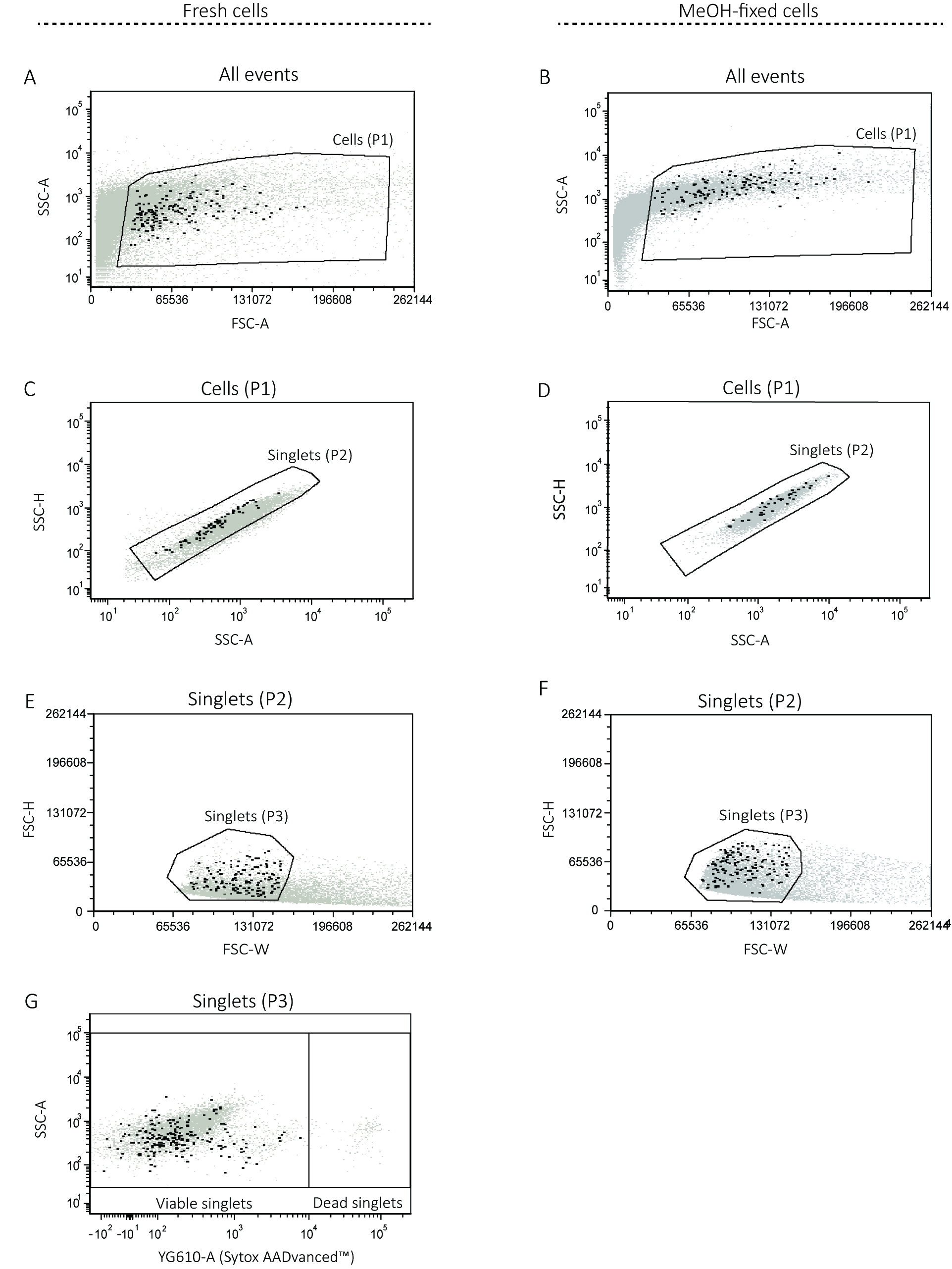

Supplement: Supplementary file 2 [file Image_1.TIF]

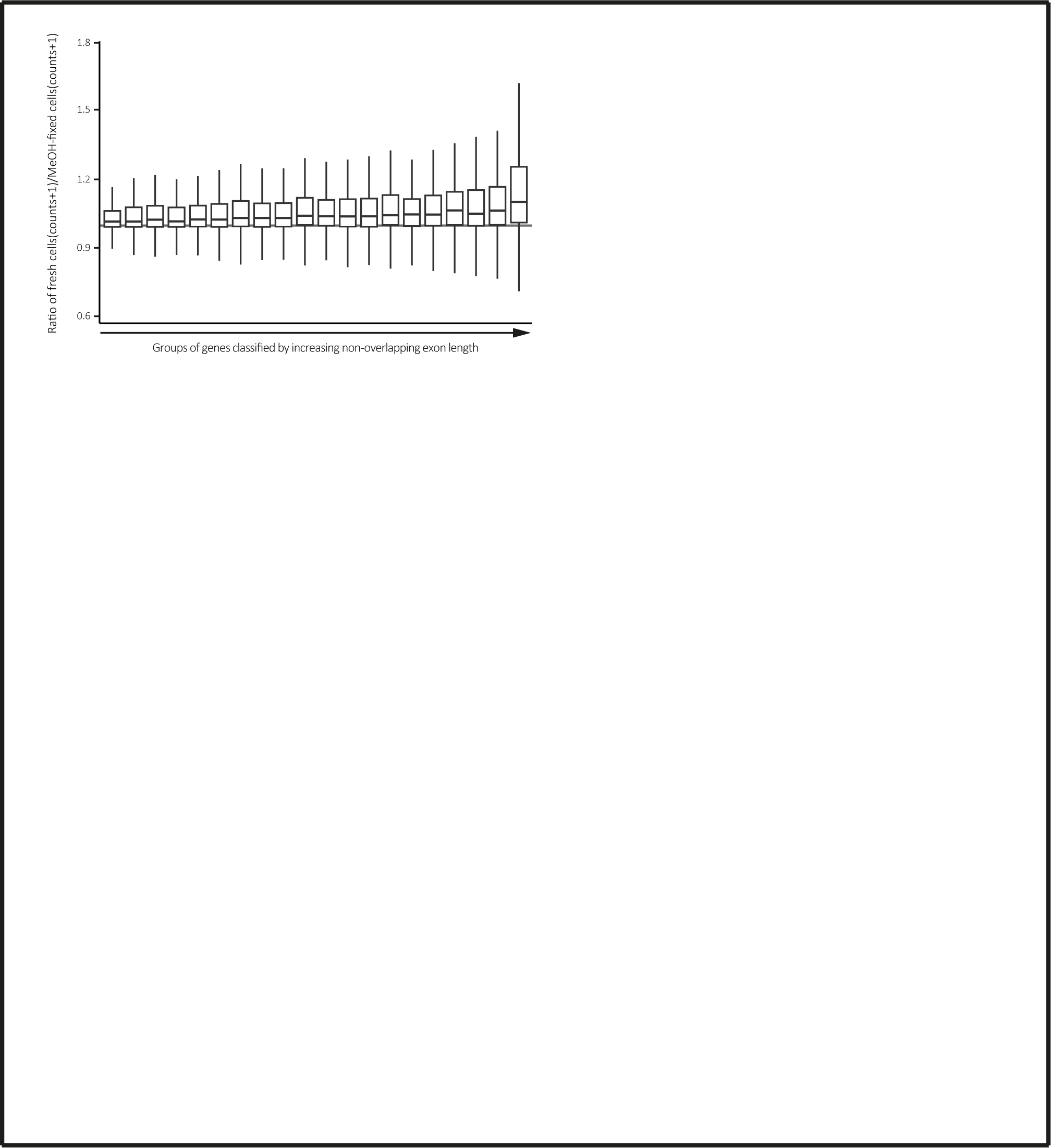

Supplement: Supplementary file 3 [file Image_2.TIF]

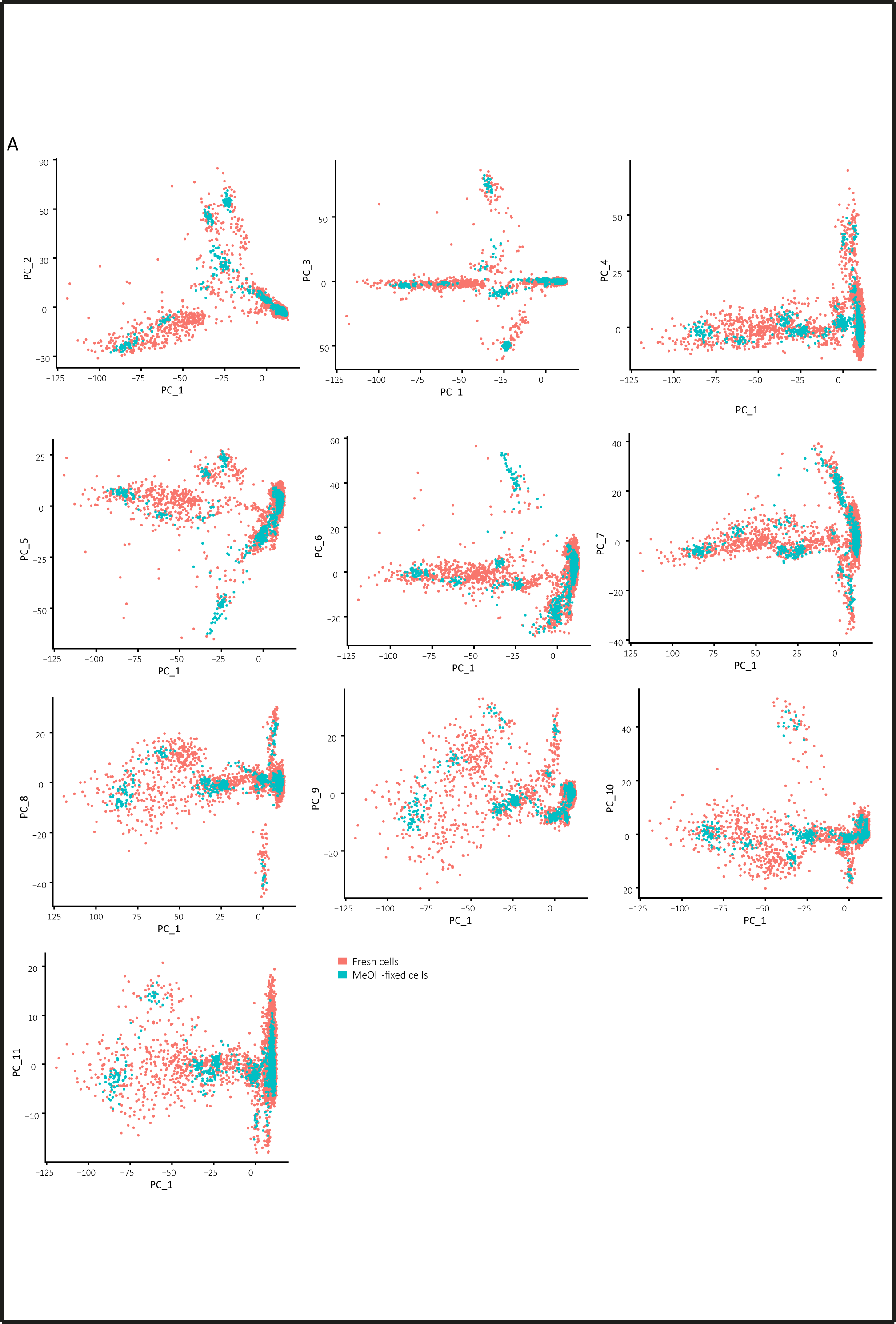

Supplement: Supplementary file 4 [file Image_3.TIF]

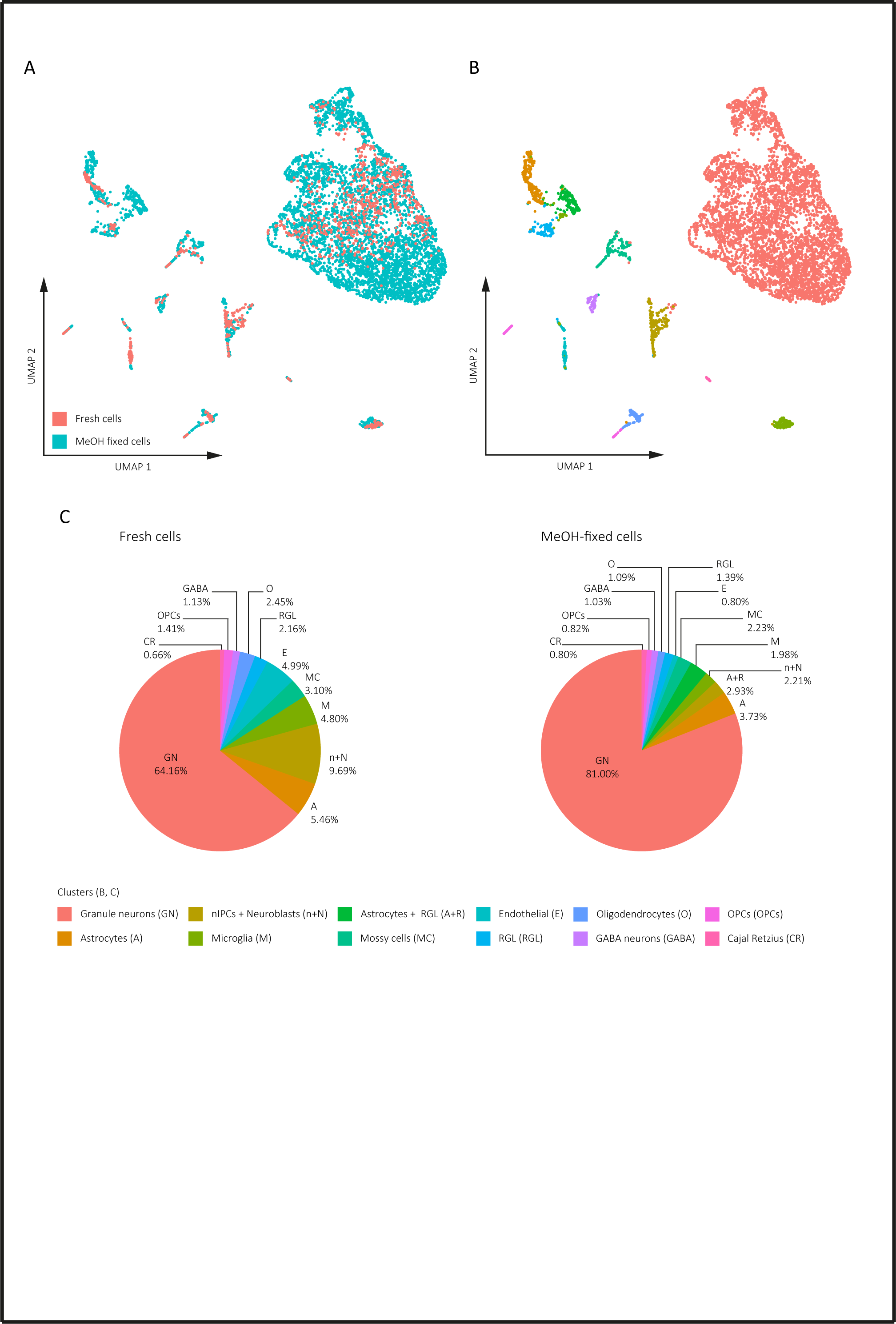

Supplement: Supplementary file 5 [file Image_4.TIF]

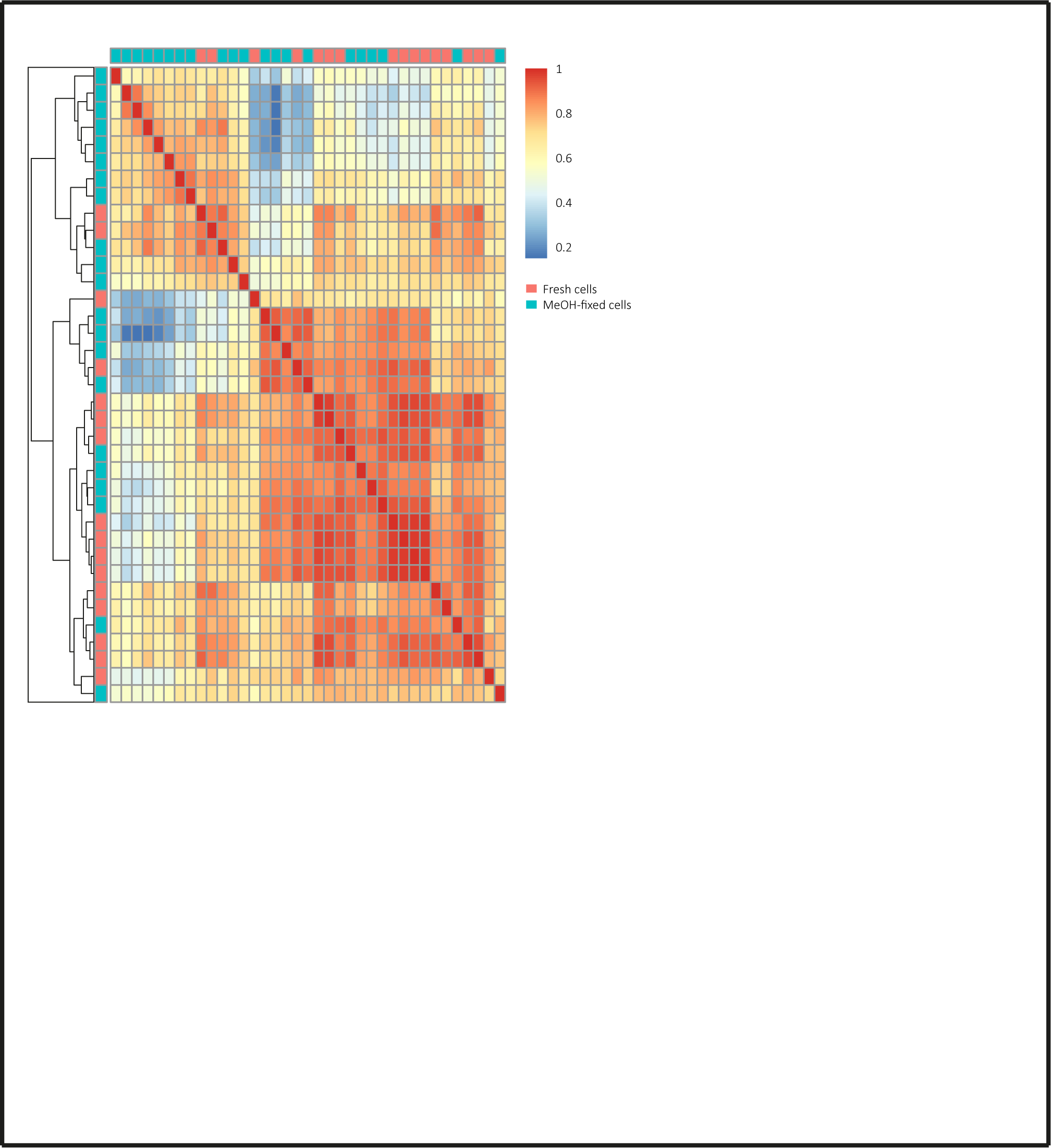

Supplement: Supplementary file 6 [file Image_5.TIF]
